# Territorial Behavior and Social Stability in the Mouse Require Correct Expression of Imprinted *Cdkn1c*

**DOI:** 10.3389/fnbeh.2018.00028

**Published:** 2018-02-26

**Authors:** Gráinne I. McNamara, Rosalind M. John, Anthony R. Isles

**Affiliations:** ^1^Behavioural Genetics Group, MRC Centre for Neuropsychiatric Genetics and Genomics, Neuroscience and Mental Health Research Institute, Cardiff University, Cardiff, United Kingdom; ^2^School of Biosciences, Cardiff University, Cardiff, United Kingdom

**Keywords:** genomic imprinting, *Cdkn1c* (*p57*^Kip2^), social group, dominance, epigenetics

## Abstract

Genomic imprinting, the epigenetic process by which transcription occurs from a single parental allele, is believed to influence social behaviors in mammals. An important social behavior is group living, which is enriched in Eutherian mammals relative to monotremes and marsupials. Group living facilitates resource acquisition, defense of territory and co-care of young, but requires a stable social group with complex inter-individual relationships. Co-occurring with increased group living in Eutherians is an increase in the number of imprinted loci, including that spanning the maternally expressed *Cdkn1c*. Using a ‘loss-of-imprinting’ model of *Cdkn1c* (*Cdkn1c*^BACx1^), we demonstrated that twofold over expression of *Cdkn1c* results in abnormal social behaviors. Although, our previous work indicated that male *Cdkn1c*^BACx1^ mice were more dominant as measured by tube test encounters with unfamiliar wild-type (WT) males. Building upon this work, using more ecologically relevant assessments of social dominance, indicated that within their normal social group, *Cdkn1c*^BACx1^ mice did not occupy higher ranking positions. Nevertheless, we find that presence of *Cdkn1c*^BACx1^ animals within a group leads to instability of the normal social hierarchy, as indicated by greater variability in social rank within the group over time and an increase in territorial behavior in WT cage-mates. Consequently, these abnormal behaviors led to an increased incidence of fighting and wounding within the group. Taken together these data indicate that normal expression of *Cdkn1c* is required for maintaining stability of the social group and suggests that the acquisition of monoallelic expression of *Cdkn1c* may have enhanced social behavior in Eutherian mammals to facilitate group living.

## Introduction

Genomic imprinting describes a subset of genes in mammals that are expressed monoallelically in a parent-of-origin specific manner as a result of epigenetic processes initiated in the germline (Ferguson-Smith, 2011). The functions of imprinted genes converge on key mammalian biological processes (Cleaton et al., 2014), including placental development, embryonic growth, and energy homeostasis and metabolism.

Imprinted genes are also important for brain development and behavior (Davies et al., 2015), with some evolutionary ideas pointing to social behaviors as being particularly targeted (Brandvain et al., 2011; McNamara and Isles, 2014). We have previously shown that animals with a loss of neural expression of the imprinted gene *Growth factor receptor bound protein 10* (*Grb10*) displayed altered social behavior. *Grb10^patKO^* males were found to be significantly more likely to win in a tube test with unfamiliar males and ‘barbered’ their cage-mates more frequently than wild-type (WT) animals (Garfield et al., 2011). More recently, we have demonstrated that a mouse model of loss-of-imprinting of the maternally expressed gene *Cyclin dependent kinase 1c* (*Cdkn1c* or *p57^Kip2^*) shows the same behavior (McNamara et al., 2017). Specifically, transgenic mice over-expressing *Cdkn1c* by twofold in the CNS were also more likely to win in a tube test with unfamiliar males. Taken together these findings have been suggested to indicate a role for imprinted genes in regulating social dominance. However, the extent to which these findings can be interpreted as imprinted genes influencing social dominance, particularly in the context of normal mouse interactions, has been questioned (Curley, 2011; comment on Garfield et al., 2011).

Numerous animal species form social groups that require nuanced social interactions to facilitate group living. In mammals, monotremes and marsupials are largely solitary whereas placental mammals, the Eutherians, have an array of social groupings (Muller and Thalmann, 2000). Group living is thought to have evolved for enhancement of fitness of individual members of the group (Alexander, 1974; Costa et al., 2012). While dominance over other animals within a group can ensure better feeding (Cordero and Sandi, 2007; Wang et al., 2011) and mating opportunities (Nelson et al., 2013, 2015), access to other reinforcing stimuli (Vargas-Perez et al., 2009) and additional health benefits (Ebbesen et al., 1992; Moles et al., 2006; Sa-Rocha et al., 2006; Golden et al., 2011), group instability (partner changes) induces anxiety and stress (Saavedra-Rodriguez and Feig, 2013) and reduces overall breeding rate in male and female in rodent species (Lardy et al., 2015).

Here, we explore the consequence of loss of imprinting of *Cdkn1c* on social dominance behavior in greater detail and in a more ecological relevant manner than our previous work (McNamara et al., 2017). Again, we utilized a murine model carrying a single extra copy of the *Cdkn1c* genomic region (*Cdkn1c*^BACx1^) on a bacterial artificial chromosome (BAC) transgene which drives entopic spatially and temporally accurate expression of *Cdkn1c* in the developing nervous system such that *Cdkn1c* is expressed at twice the normal level, effectively mimicking loss of imprinting (John et al., 2001; Andrews et al., 2007). We also make use of a control reporter line carrying the same BAC transgene but with transgenic expression of *Cdkn1c* replaced by β-galactosidase (*Cdkn1c*^BACLacZ^). These control transgenic mice have WT *Cdkn1c* expression levels (John et al., 2001; Andrews et al., 2007; Tunster et al., 2010) and serve as a reference for efficacy of testing procedures. Phenotypes present in the *Cdkn1c*^BACx1^ transgenic line and absent in the *Cdkn1c*^BACLacZ^ transgenic line can therefore be attributed to increased expression of *Cdkn1c* alone (McNamara et al., 2016, 2017). Using an array of ecologically relevant tests (tube test; scent marking; competition for a limited resource; in-cage fighting) that provide converging evidence for levels of social dominance (Wang et al., 2011), we show that *Cdkn1c*^BACx1^ mice are not more socially dominant *per se*, but that correct *Cdkn1c* imprinting and expression is critical for the stability of group social structure.

## Materials and Methods

### Animals and Signs of Fighting

All procedures were conducted in accordance with the requirements of the United Kingdom Animals (Scientific Procedures) Act 1986, under the remit of Home Office license number 30/2673. These procedures were also approved by the appropriate ethics committee at Cardiff University.

The experimental mouse line, *Cdkn1c*^BACx1^ possesses one copy of a BAC that spans the *Cdkn1c* gene and two other genes, *Phlda2* and *Slc22a18*. *Cdkn1c*^BACx1^ were compared to their WT cage-mates in all instances. A separate reporter line *Cdkn1c*^BACLacZ^ possesses a modified version of this BAC with a β-galactosidase reporter construct inserted into the *Cdkn1c* locus, disrupting *Cdkn1c* expression (John et al., 2001; Andrews et al., 2007). *Cdkn1c*^BACLacZ^ were compared to their WT cage-mates in all instances.

Male mice were group housed from weaning at 3–4 weeks, with between three and five animals per cage. Each cage consisted of transgenic animals (*Cdkn1c*^BACx1^ or *Cdkn1c*^BACLacZ^) and WT litter-mates. All were housed in a 12:12 h light:dark cycle with food and water provided *ad libitum* except during the “competition for water access test” where all animals had restricted access to water, provided for 2 h per day, immediately following behavioral testing.

Coat condition and general appearance was monitored regularly from weaning. Occurrences of injury due to bullying/fighting (fresh wounds on the flanks or in the ano-genital region) were recorded in both the behavioral cohort and the stock cohort, to maximize observational size.

Animals were between 8 and 12 weeks at beginning of testing. For all experiments *N* = 48 animals were used in total: *Cdkn1c*^BACx1^ (*n* = 14) and their WT cage-mates (*n* = 12); *Cdkn1c*^BACLacZ^ (*n* = 15) and their WT cage-mates (*n* = 8). Average number of animals per cage: *Cdkn1c*^BACx1^ cohort = 3.9 SEM = 0.3 (7 cages); *Cdkn1c*^BACx1^ cohort = 3.8 SEM = 0.4 (6 cages). Test order was as follows; within-cage tube test, scent marking task and water access task. Following these experiments animal’s rank stability with and without a bedding change was assessed. For analysis of within-cage measures of dominance, bedding was unchanged for the duration of each task but was changed between each task to allow for a more representative indication of rank stability over time.

### Tests of Social Behaviors

#### Tube Test

The tube test was carried out as previously described (Garfield et al., 2011; McNamara et al., 2017). Briefly, the test apparatus consisted of a 30 cm, transparent tube with a 3.5 cm diameter placed in an opaque arena to obscure view of the environment. Testing was carried out in dimmed light conditions. At the beginning of each trial two animals were introduced into the tube from both ends and released simultaneously. A trial was complete when one animal fully backed out of the tube. The animal that did not back out was considered the dominant animal of the trial. This task has been used routinely to assess dominance in dyadic pairings, with variable correlation with other measures of dominance reported in the literature (Lindzey et al., 1961).

##### Within cage

All encounters in the tube test were within-cage (i.e., with cage-mates). For the duration of the experiment the home cage bedding remained unchanged. To exclude the effect of anxiety to a novel environment, animals were trained individually to pass through the tube for 2 days prior to testing. On test days all animals faced each of its cage mates in a ‘round robin’ design and this was carried out for four consecutive days. As such, for a cage size of n, each animal had n-1 encounters per day. Side of entry to tube was counterbalanced by day. Pilot studies on a 10 day experiment indicated no substantial difference between rank across 4 consecutive days and across 10 consecutive days. 4 consecutive days was chosen for welfare reasons as, including training days, this represented 6 days without a cage clean. For each day an animal was given a rank depending on the number of encounters won, the animal that didn’t back down in any of its trials was designated the most dominant animal in the cage and was given a rank of 1. The most subordinate animal in the cage was designated as the animal that backed down before each of its opponents and was given the lowest rank (0), and so on for each animal in the cage. For example, in a cage of three individuals, the assigned ranks would be 1 (‘alpha’), 0.5 (‘beta’), and 0 (‘gamma’). This allowed us to compare across cages differing in total number. While this lacks some sensitivity regarding the animals just above the lowest rank or below the highest rank, this would be sensitive to a large group effect size.

After 4 days each animal had an average rank score. *Cdkn1c*^BACx1^ (*n* = 12) and their WT cage-mates (*n* = 11); *Cdkn1c*^BACLacZ^ (*n* = 15) and their WT cage-mates (*n* = 8). In this task one cage (*n* = 4) was excluded, as one individual in the group was too large to pass through the tube freely.

##### Environment change

This experiment followed the same protocol as the within cage tube test. Animals’ rank was assessed in four sessions; on day 1, to exclude effects of novelty (E1), day 2 in the morning 1 h prior to bedding change (E2) and in the afternoon (E3) and day 3 in the afternoon (E4). Between E2 and E3 the home cage was cleaned, animals were moved to a new cage and all bedding was replaced to remove any odors identifying the previously dominant animal in the group. E3 and E4 were carried out 1 and 24 h, respectively, after cage change. For statistical analysis, whether an animals’ rank differed over an environment change (E2 to E3) compared to when there was no change in the environment (E3 to E4) was recorded as ‘0’ for no change in rank and ‘1’ for rank changed, regardless of the direction of the change. Specifically, if an animal had a rank of 3 before the bedding change and 4 after, this was recorded at 1. If an animal had a rank of 4 before the bedding change and 4 after, this was recorded as 0. The average score per genotype means that the closer to 1 for a genotype, the closer to 100% of animals changed rank and vice versa (e.g., 0+1+0+1+0+1+0+1+0+1 = 5/10 = 50% of animals changed rank. *Cdkn1c*^BACx1^ (*n* = 12) and their WT cage-mates (*n* = 11); *Cdkn1c*^BACLacZ^ (*n* = 15) and their WT cage-mates (*n* = 8).

#### Scent Marking

For this experiment an arena 30 cm × 30 cm × 30 cm was bisected by a wire mesh (grid size 0.6 cm × 0.6 cm). Both sides of the floor were lined with absorbent paper (3MM Whatman, Fisher Scientific). Each encounter consisted of one animal and a cage-mate placed on either side of the wire mesh, through which they could receive visual, auditory and olfactory information, but could not physically interact. The experiment was carried out under dim lighting conditions and each encounter lasted 1 h. Each individual animal met each of its cage-mates in such an encounter, with no animal having more than one encounter per day. Scent marks made on absorbent filter paper were visualized under ultraviolet light and outlined by pencil. Analysis was modified from (Arakawa et al., 2007). A grid of 1 cm × 1 cm squares (420 squares in total) was overlaid and the number of squares containing scent marks was recorded for each animal for each encounter. Marks greater than 4 squares in size were excluded in order to differentiate general urine pools from specific scent marks. The dominant animal in the encounter was designated as the animal that scent marked more than its opponent. Each animal was assigned a rank depending on the number of encounters ‘won’ in this manner. *Cdkn1c*^BACx1^ (*n* = 15) and their WT cage-mates (*n* = 12); *Cdkn1c*^BACLacZ^ (*n* = 15) and their WT cage-mates (*n* = 8).

#### Competition for Water Access

During the experiment animals had restricted access to water (see above) and were individually trained to locate and consume freely available water, provided though a metal drinking spout in a 600s session in a Phenotyper arena (Noldus Information Technology). Within three daily training sessions all animals had successfully learnt that water was available as indexed by visual confirmation of initiation of water consumption within 30 s of drinking spout presentation. Following training, a test session was performed in which *all* animals from a given cage group were placed in the Phenotyper arena and the same time and drinking spout was introduced. The session was digitally recorded and order and ID of each animal’s introduction to the arena was recorded and used for identification during scoring. The duration each animal spent drinking was then scored manually offline. Each animal was assigned a rank depending on the duration of water access obtained in the first 120 s and the full 600 s. The most dominant animal in the cage was the animal that had the greatest duration of water access, the ‘beta’ animal having the second highest amount of access, and so on. *Cdkn1c*^BACx1^ (*n* = 14) and their WT cage-mates (*n* = 12); *Cdkn1c*^BACLacZ^ (*n* = 15) and their WT cage-mates (*n* = 8). One individual died between scent marking and competition for water access tasks.

### Olfactory Function

Two separate tests of olfactory function were used, one using social odors and another using non-social odors.

#### Social Odor

Animals were allowed to habituate in an open field arena (300 mm × 300 mm, and illuminated evenly with a 60 W bulb) for 120 s in the absence of any odor, then returned to their home-cage. Absorbent filter paper was scented with 20 μl of fresh male urine and placed under a permeable cover. Animals were then returned to the arena, with the odor in place. Activity was tracked using a camera connected to a computer with ETHOVISION software (Noldus, Nottingham, United Kingdom) and time spent investigating the odor (defined to be when the middle of the animal was within 1 cm of the odor) was analyzed.

#### Food Odor

Arena set up as above with the addition of 2 cm of clean sawdust on base of arena. A cookie was submerged under sawdust. Animals were placed in the opposite quadrant to the cookie and the quadrant was changed for each successive animal. Activity was tracked using a camera connected to a computer with ETHOVISION software (Noldus, Nottingham, United Kingdom) and latency to sniff the cookie (defined to be when the middle of the animal was within 1 cm of the odor) was recorded digitally. In addition, latency to find and begin eating the cookie was recorded manually. Trials ended once animal began to eat cookie.

### Statistical Analysis

All statistical analysis was carried out using SPSS 20.0 (SPSS, United States). For analysis of genotype on rank within a cage group, each animal’s rank within the cage was transformed to a number between 0 (least dominant animal in the group) and 1 (most dominant animal in the group), this was performed for each group for each task to allow for differences in cage group size. A non-parametric Mann–Whitney *U*-test was carried out with GENOTYPE as the grouping variable.

Rank stability across a change in environment (cage-bedding change) or when the environment remained stable was rated as ‘0’ for no change and ‘1’ for change in rank in a tube test. Effect of GENOTYPE was assessed using a conditional logistic regression, regressing on CAGE ID, to take into account cage group sizes and account for extreme cages. Scent marking behavior was analyzed using a linear mixed-models ANOVA, with CAGE ID as the random factor; pairwise fixed effects were then assessed by Bonferroni. Correlation between ranks within groups across difference tasks was determined using a non-parametric Spearman’s rank-order correlation and statistical difference between correlation coefficients was assessed using Fisher r-to-z transformation. For analysis of effect of genotype on likelihood to be involved in severe fighting, a chi-square cross-tabs test was carried out with GENOTYPE as rows and BITE PRESENCE as column. Olfactory function analysis was carried out using a repeated measures ANOVA with GENOTYPE as the between subject measure and ODOR PRESENCE and the within subject measure.

## Results

### A Stable Hierarchy Is Disrupted in the Presence of a *Cdkn1c*^BACX1^ Male

**FIGURE 1:**
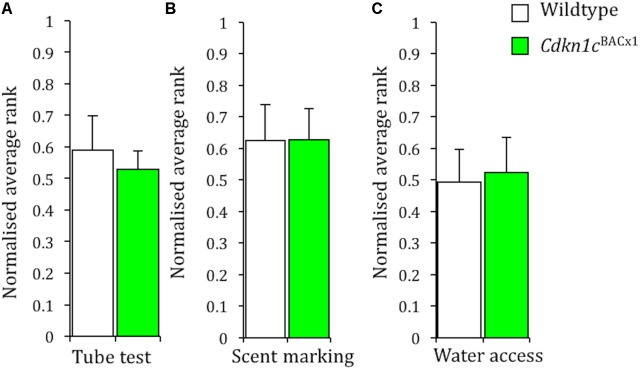
*Cdkn1c* over expression does not affect dominance behaviors within the home cage group. There was no effect of GENOTYPE on the average group rank in the within-cage tube test **(A)**, the scent marking task **(B)**, and the water access task **(C)**. Data shown are means ± SEM.

Group housed male mice establish a linear, transitive (Williamson et al., 2016), social hierarchy with a single dominant individual and a number of sequential (beta, gamma, delta) subordinates (Ebbesen et al., 1992; Avitsur et al., 2007; Wang et al., 2011). We carried out three tasks assessing social dominance (tube test, urine marking, and competitive access to a resource) to determine an animal’s rank within its cage group. Strikingly, given our previous findings (McNamara et al., 2017), *Cdkn1c*^BACx1^ animals did not occupy significantly more dominant ranks than their WT cage-mates on any individual measure of the within cage social hierarchy. Specifically, there was no difference in social rank as determined by the tube test (**Figure 1A**; Mann–Whitney, *U* = 51, *p* = 0.59), scent marking (**Figure 1B**; *U* = 42.5, *p* = 0.27) or competitive water access (**Figure 1C**; 120 s: *U* = 49.5, *p* = 0.51; data not shown, 600 s: *U* = 43.5, *p* = 0.29).

The *Cdkn1c*^BACLacZ^ control line, where *Cdkn1c* was not overexpressed, was also subjected to the same battery of social dominance tests and here too there was no difference in social rank between these males and their WT cage-mates (Scent marking: *U* = 48, *p* = 0.47; Water access 120 s: *U* = 54, *p* = 0.73; Water access 600 s: *U* = 50, *p* = 0.55; **Supplementary Figure S1**). These results are not caused by an inability to perform the tasks, as a clear transitive hierarchy was apparent in each measure of social dominance. For instance, a linear, transitive, hierarchy was apparent for an average of 3.4/4 days groups of *Cdkn1c*^BACLacZ^ animals and their cage mates, and an average 3.3/4 days in groups of *Cdkn1c*^BACx1^ animals and their cage mates. Similarly, a clear hierarchy was apparent in 100% of cages containing *Cdkn1c*^BACLacZ^ animals and in 85.7% of cages containing *Cdkn1c*^BACx1^ animals in the competition for water access task. A clear hierarchy was also apparent in 71.4% cage of cages containing *Cdkn1c*^BACLacZ^ animals and in 57.1% of cages containing *Cdkn1c*^BACx1^ animals in the scent marking task.

**FIGURE 2:**
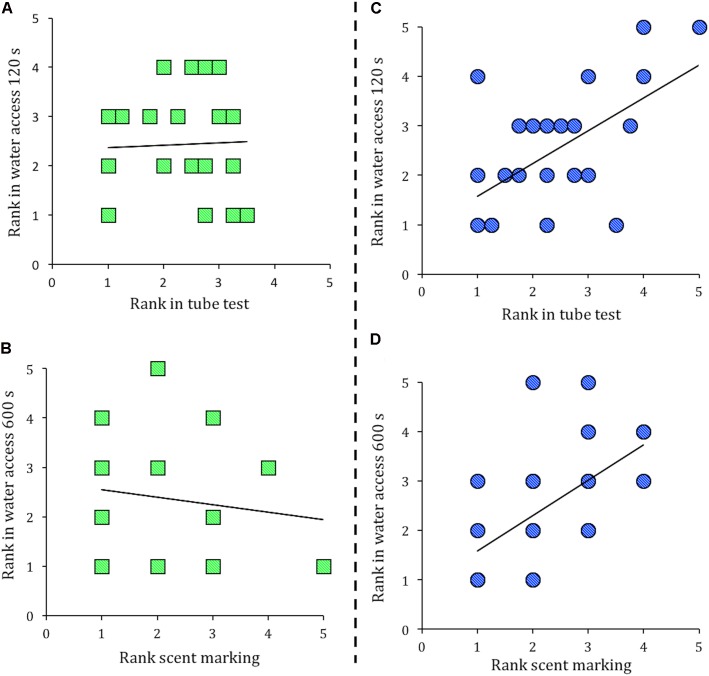
Presence of a *Cdkn1c*^BACx1^ male destabilizes the established social hierarchy. In groups containing *Cdkn1c*^BACx1^ males and their wild-type (WT) cage-mates there was no correlation between rank in the tube test and rank in the water access task in the first 120 s **(A)**, nor rank in the scent marking task is correlated with rank in the water access task in 600 s **(B)**. In cages of *Cdkn1c*^BACLacZ^ and their WT cage-mates, an animal’s rank in the tube test correlated with rank in the water access task in the first 120 s **(C)**. Additionally, rank in the scent-marking task correlated with rank in the water access task in 600 s **(D)**.

In a stable social hierarchy an individual’s rank in these separate measures is expected to correlate (Wang et al., 2011). However, in groups of *Cdkn1c*^BACx1^ and their WT cage-mates the social hierarchy was not stable, as an individual’s rank in one measure of dominance did not correlate with its rank in another (**Figure 2A**, tube test vs. water access in the first 120 s rank, Spearman’s ρ correlation = -0.034, *p* = 0.88; and **Figure 2B**, scent marking vs. water access 600 s rank, Spearman’s ρ correlation = -0.134, *p* = 0.51). In contrast, groups containing the control line *Cdkn1c*^BACLacZ^ animals and their WT cage-mates showed the expected pattern, as an individual’s rank in one measure of dominance was significantly correlated with its rank in another measure. Specifically, tube test vs. water access in the first 120 s rank (**Figure 2C**; Spearman’s ρ correlation = 0.521, *p* = 0.01) and scent marking vs. water access 600 s rank (**Figure 2D**; Spearman’s ρ correlation = 0.665, *p* = 0.001). Fisher r-to-z transformations confirmed these group differences, as the correlation coefficients seen in groups of *Cdkn1c*^BACx1^ and their WT cage-mates were significantly different from the correlation coefficients between in groups of *Cdkn1c*^BACLacZ^ animals and their WT cage-mates (Tube test vs. water access 120 s, *z* = 1.93, *p* = 0.05; Scent marking vs. water access 600 s, *z* = 3.06, *p* = 0.002).

### Rank of *Cdkn1c*^BACX1^ Mice Varies More Frequently Than Wild-Type Cage-Mates

**FIGURE 3:**
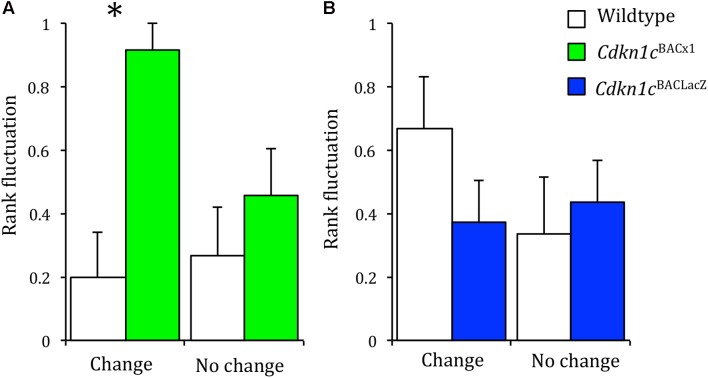
When odor cues indicating dominance are removed, *Cdkn1c*^BACx1^ males change rank more frequently than WT. In groups of *Cdkn1c*^BACLacZ^ and WT animals, rank fluctuation did not differ when olfactory cues indicating the dominant animal were removed (**A**, left) nor when the environment remained stable (**A**, right). When odor cues were removed *Cdkn1c*^BACx1^ males’ rank fluctuated significantly more than WT animals (**B**, left). This was not the case when the environment remained stable (**B**, right). Data shown are means ± SEM. ^∗^*p* < 0.05.

We hypothesized that the loss of stability between different measures of social dominance may be as a consequence of a greater propensity of *Cdkn1c*^BACx1^ animals to challenge the established hierarchy. Therefore, we would expect these animals to have a more variable rank in the cage hierarchy across time. Dominancy relationships, while generally stable, can change under pressurizing circumstances (Cohn et al., 2012). One such circumstance is when the odor cues are removed (e.g., following a bedding change), after which the hierarchy must be re-established (Gray and Hurst, 1995; Van Loo et al., 2000). However, if the social structure is generally stable, the change of bedding should not perturb an animal’s rank any greater than in an unchanging environment. We examined this using consecutive tube tests, comparing an animal’s rank before and after odor cues were removed (a cage-bedding change). When odor cues remained constant, there was no greater change in the rank of *Cdkn1c*^BACx1^ animals or their WT cage-mates across repeated testing (Wald statistic = 0.021, *p* = 0.886). However, when odor cues were removed following a cage-bedding change, *Cdkn1c*^BACx1^ animals had a significantly more variable rank compared to their WT cage-mates (Wald statistic = 3.925, *p* = 0.048) (**Figure 3A** and **Supplementary Table S1**).

In the control group, both *Cdkn1c*^BACLacZ^ and their WT cage-mates also displayed no difference in change in rank when the environment remained stable (Wald statistic = 0.010, *p* = 0.922; **Figure 3B** and **Supplementary Table S2**). However, in contrast to the *Cdkn1c*^BACx1^ animals, the *Cdkn1c*^BACLacZ^ mice also showed no difference in change in rank following a bedding change (Wald statistic = 0.665, *p* = 0.415). This indicates that in the absence of odor communicants indicating the dominant animal *Cdkn1c*^BACx1^ animals are more likely to change rank.

### Territorial Marking Is Moderately Changed in Response to *Cdkn1c*^BACx1^ Males

**FIGURE 4:**
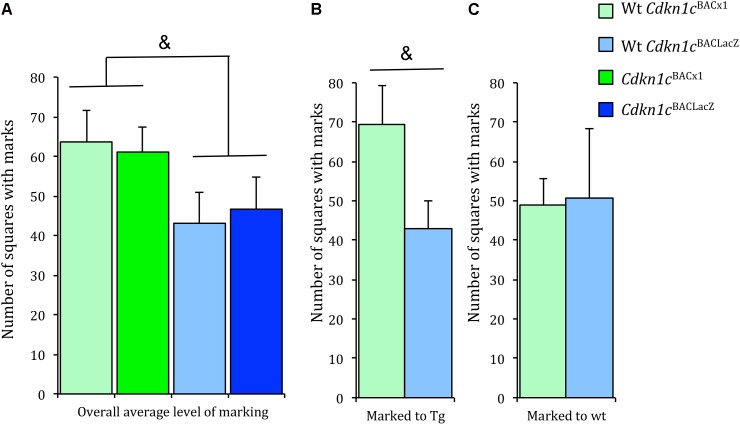
Wild-type animals increased scent marking toward *Cdkn1c*^BACx1^ but not *Cdkn1c*^BACLacZ^ animals. **(A)** There were greater levels of scent marking in *Cdkn1c*^BACx1^ containing groups compared to *Cdkn1c*^BACLacZ^ containing groups. **(B)** WT cages mates of *Cdkn1c*^BACx1^ animals scent marked more to transgenes (tg) than **(C)** WT cages mates of *Cdkn1c*^BACLacZ^ animals. Data shown are means ± SEM. ^&^*p* = 0.07, ^∗^*p* = 0.05.

The maintenance of social status within a specific territory is an important aspect of social behavior. Both wild-caught and laboratory mice establish territories (Anderson and Hill, 1965; Hurst, 1987; Fuxjager et al., 2010). Territorial ownership is communicated via ‘scent marks’ composing of major urinary proteins (Hurst et al., 1993, 2001). This scent marking behavior positively influences male reproductive success (Thonhauser et al., 2013), demonstrating a clear advantage upon which selective pressures may act. Scent marking communicates a dominant animal’s territory and scent-marking activity is altered in the presence of a dominant resident (Drickamer, 2001). An general assessment of the scent marking test indicated approximately 30% increase in the level of scent marking by *Cdkn1c*^BACx1^ animals and their WT cage-mates in comparison to *Cdkn1c*^BACLacZ^ animals and their WT cage-mates, although this failed to reach significance [*t*(54) = -1.85, *p* = 0.07] (**Figure 4A**). A more detailed examination of these data revealed that WT animals increased scent marking toward (i.e., when paired with) a *Cdkn1c*^BACx1^ male, compared to a control *Cdkn1c*^BACLacZ^ male [*t*(19) = 1.96, *p* = 0.074] (**Figure 4B** and **Supplementary Figure S3**) or toward another WT cage-mate [*t*(13) = 0.006, *p* = 0.94] (**Figure 4C** and **Supplementary Figure S3**). This indicates that the presence of *Cdkn1c*^BACx1^ animals may elicit a differential territorial behavior response in WT cage-mates.

### Olfactory Function in *Cdkn1c*^BACx1^ Is Normal

**FIGURE 5:**
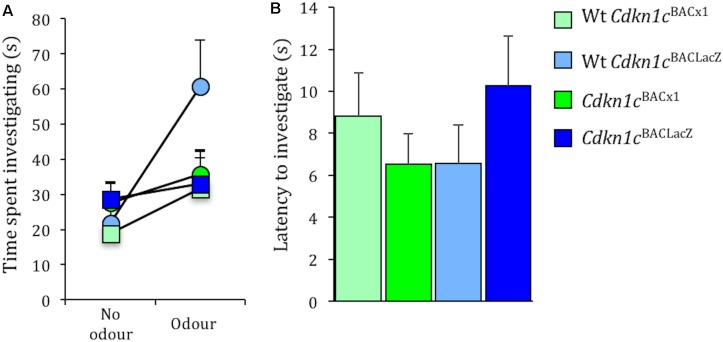
Olfactory response to social and non-social odors is normal in *Cdkn1c*^BACx1^ mice. There was no difference in time spent exploring a social odor **(A)**. Latency to detect a non-social odor was similar between all groups **(B)**.

Importantly, the effects on social stability are unrelated to general olfactory function *per se*, as there was no difference between *Cdkn1c*^BACx1^ and WT animals in time spent exploring a social odor (male urine) [*t*(24) = -0.29, *p* = 0.79, no odor present; *t*(24) = -1.41, *p* = 0.18, odor present] (**Figure 5A**). Similarly there was no difference between *Cdkn1c*^BACx1^ and WT animals in ability to detect a non-social odor (food) [latency to sniff: *t*(22) = -0.783, *p* = 0.44] (**Figure 5B**).

### An Unstable Social Environment Has Consequences for Fitness

A stable social hierarchy normally benefits group-housed animals (Ebbesen et al., 1992; Moles et al., 2006; Sa-Rocha et al., 2006; Cordero and Sandi, 2007) and the more frequent rank fluctuation of *Cdkn1c*^BACx1^ animals appeared to have a consequence for fitness. We observed significantly more signs of severe in-cage fighting (fresh cuts along flanks or in ano-genital region observed on at least one occasion) in cages containing animals over-expressing *Cdkn1c* and their WT cage-mates (42% of animals, *n* = 50) compared to cages of *Cdkn1c*^BAClacZ^ animals and their WT cage-mates (no observed occurrences, *n* = 56) and cages containing only WT litter-mates of *Cdkn1c*^BACx1^ animals (no observed occurrences, *n* = 7). Within groups of *Cdkn1c*^BACx1^ and their WT cage-mates signs of severe fighting were not different by genotype (Fischer’s exact test, *p* = 0.26; **Supplementary Figure S2**), indicating that presence of a *Cdkn1c*^BACx1^ in a group had negative effects on physical fitness for all animals within the social group.

## Discussion

The correct expression of imprinted genes is critical for a number of aspects of physiology (Cleaton et al., 2014). Here, using a transgenic BAC mouse model (*Cdkn1c*^BACx1^), we demonstrated that twofold over expression of *Cdkn1c* results in abnormal social behaviors. Although our previous work indicated that male *Cdkn1c*^BACx1^ mice were more dominant as measured by tube test encounters with unfamiliar WT males (McNamara et al., 2017), a detailed and more ecologically relevant assessment of social dominance suggested that within their normal social group, *Cdkn1c*^BACx1^ mice did not occupy higher ranking positions. However, we find that presence of *Cdkn1c*^BACx1^ animals within a group leads to instability of the normal social hierarchy, as indicated by greater variability in social rank within the group over time and an increase in territorial behavior in WT cage-mates. These abnormal behaviors led to an increased incidence of fighting, and suggest that normal expression of *Cdkn1c* is required for maintaining stability of the social group.

In contrast to our previous finding that *Cdkn1c*^BACx1^ mice were more successful in the tube test when paired with unfamiliar mice (McNamara et al., 2017), when faced with a familiar cage mate in the same task, *Cdkn1c*^BACx1^ animals did not display an increased likelihood of a successful outcome. Two additional tests of social dominance behavior, competition for resource and a test of scent marking, confirmed this finding showing that *Cdkn1c*^BACx1^ animals were no more likely occupy the top rank position in the cage hierarchy than WT cage-mates. These three separate tests did reveal that groups containing one or more *Cdkn1c*^BACx1^ had a much less stable dominance hierarchy. Specifically that the rank of an individual changed between test-days, as indicated by an absence of correlation between the three different measures. In contrast, and as is expected (Wang et al., 2011), there was a strong correlation between the ranking derived from the tube test, urine marking and competition for resource tests in groups from the control group, made up of WT and *Cdkn1c*^BACLacZ^ males. Importantly, the lack of a correlation between measures in cage of *Cdkn1c*^BACx1^ and WT cage mates was not caused by an inability to perform the tasks, as a clear transitive hierarchy was apparent in each measure of social dominance.

To test whether the stability of dominancy in a social group is disrupted by the presence of mice over-expressing *Cdkn1c*, we carried out a further manipulation. If the social structure is generally stable, a change of environment should not have a substantial effect on an animal’s position in the social group, which was the case for cages containing males from the control line, *Cdkn1c*^BACLacZ^. In contrast, *Cdkn1c*^BACx1^ animals were significantly more likely to change rank after an environment change. The decreased stability of the social group caused by the presence of a *Cdkn1c*^BACx1^ animal also induced changes in WT cage-mate territorial behavior, as indicated by an increase in magnitude of scent marking toward *Cdkn1c*^BACx1^ animals but not control transgenic *Cdkn1c*^BACLacZ^. This was not statistically significant and repetition with a larger cohort size may provide further insight. It not possible, using these experiments to conclude definitively the origin of the disruption of the social hierarchy, and this deficit may not necessarily manifest exclusively in social behavior. Nonetheless, these direct, and indirect, actions of elevated *Cdkn1c* expression on territorial behaviors and social stability may underlie the observed increased incidence of signs of in-cage fighting.

Group living is enriched in both frequency of observation and complexity in Eutherian mammals in comparison to monotremes and marsupials (Muller and Thalmann, 2000). This has occurred in conjunction with an expansion in neocortical neuron number (Cheung et al., 2010) and an increase in connectivity (Krubitzer, 1998). A comparison of marsupials, rodents and primates found that a larger brain size was associated with social play prevalence, across taxa (Iwaniuk et al., 2001). Concurrent with this expansion of neocortical complexity and social play is the emergence of genomic imprinting, and a potentially function role for imprinted genes and the change in neocortical organization has been posited (Keverne et al., 1996). Monoallelic expression of *Cdkn1c* and the differential methylation of the CpG island encompassing its imprinting control region also emerge at this time (Suzuki et al., 2005; Ager et al., 2008) and, like a number of imprinted genes (Mercer et al., 2009; Yashiro et al., 2009; Adnani et al., 2015), *Cdkn1c* has been implicated in neocortical development and cortical function (Itoh et al., 2007; Tury et al., 2011; Colasante et al., 2013). This suggests a functional role for acquisition of monoallelic expression of *Cdkn1c* in neocortical expansion and group living, in Eutherian mammals.

Social behaviors have long been a suggested site at which genomic imprinting may exert influence (Haig, 2000; Brandvain et al., 2011; McNamara and Isles, 2014). This study provides further clear evidence in support of this idea generally but indicates that, at least for *Cdkn1c*, this is not due to effects on social dominance *per se*. Instead the findings presented here indicate a role for *Cdkn1c* in the maintenance of a cohesive social unit. Moreover, whilst further work is required, when coupled with the previous findings for *Grb10* (Garfield et al., 2011), these data suggest a substantial role of genomic imprinting in the regulation of social behavior to facilitate group living.

## Availability of Data and Material

The datasets during and/or analyzed during the current study are available from the corresponding author on reasonable request.

## Author Contributions

GMN performed the experiments and analyzed the data. GMN, RJ, and AI designed the experiments. RJ provided the materials. GMN and AI wrote the manuscript.

## Conflict of Interest Statement

Since September 2017, GMN has been employed by Frontiers Media SA. GMN declared her affiliation with Frontiers, and the handling Editor states that the process nevertheless met the standards of a fair and objective review. The other authors declare that the research was conducted in the absence of any commercial or financial relationships that could be construed as a potential conflict of interest.

## Supplementary Material

FIGURE S1 Mice from the control transgenic line, *Cdkn1c*^BACLacZ^, were no more likely to occupy a top-rank position than their wild-type cage-mates. There was no effect of GENOTYPE on the average group rank in the within-cage tube test **(A)**, the scent marking task **(B)**, and the water access task **(C)**. Data shown are means ± SEM.

FIGURE S2 Increased frequency of bite-marks and injuries from fighting in mice in groups containing one or more *Cdkn1c*-overexpressing mice. There were significantly more signs of severe in-cage fighting (fresh cuts along flanks or in ano-genital region observed on at least one occasion) in cages containing animals over-expressing *Cdkn1c* and their wild-type cage-mates compared to cages of *Cdkn1c*^BAClacZ^ animals and their wild-type cage-mates. Within groups of *Cdkn1c*^BACx1^ and their wild-type cage-mates signs of severe fighting were not different by genotype.

FIGURE S3 Wild type animals increased scent marking toward *Cdkn1c*^BACx1^ but not *Cdkn1c*^BACLacZ^ animals. Average marking per individual to by a wt cagemate of *Cdkn1c*^BACx1^ (green) or a wt cagemate of *Cdkn1c*^BACLacZ^ (blue) to either a wt or tg cagemate.

TABLE S1 Provides the within-cage rank as determined by the tube test for *Cdkn1cBACx1* and WT litter mates. Individual data before and after a bedding change is indicated and the “rank change score” also provided.

TABLE S2 Provides the within-cage rank as determined by the tube test for *Cdkn1cBACLacZ* and WT litter mates. Individual data before and after a bedding change is indicated and the “rank change score” also provided.
