# Supplementary material for: Territorial Behavior and Social Stability in the Mouse Require Correct Expression of Imprinted Cdkn1c
Source: Front Behav Neurosci. 2018 Feb 26;12:28. doi: 10.3389/fnbeh.2018.00028 (PMC5834910; doi:10.3389/fnbeh.2018.00028)
Supplement: FIGURE S1 — Mice from the control transgenic line, Cdkn1cBACLacZ, were no more likely to occupy a top-rank position than their wild-type cage-mates. There was no effect of GENOTYPE on the average group rank in the within-cage tube test (A), the scent marking task (B), and the water access task (C). Data shown are means ± SEM. [file Image_1.PDF]

Territorial behaviour and social stability in the mouse require correct expression of imprinted *Cdkn1c*

Gráinne I. McNamara, Rosalind M. John & Anthony R. Isles.

Figure S1

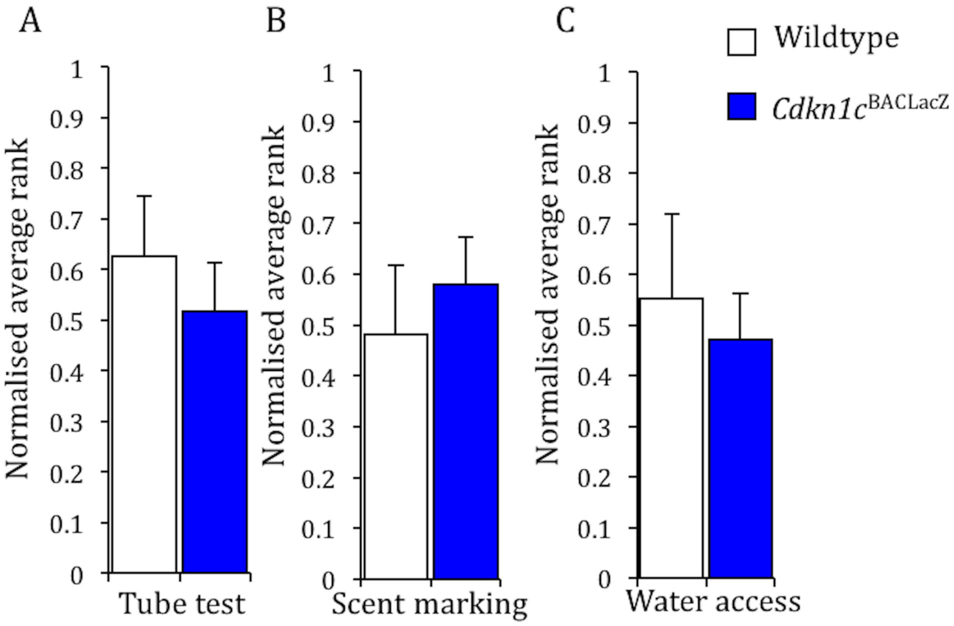

Figure S1 Mice from the control transgenic line, *Cdkn1c*^{BACLacZ}, were no more likely to occupy a top-rank position than their wild type cage-mates. There was no effect of GENOTYPE on the average group rank in the within-cage tube test (A), the scent marking task (B) and the water access task (C). Data shown are means ± SEM
