# Supplementary material for: Territorial Behavior and Social Stability in the Mouse Require Correct Expression of Imprinted Cdkn1c
Source: Front Behav Neurosci. 2018 Feb 26;12:28. doi: 10.3389/fnbeh.2018.00028 (PMC5834910; doi:10.3389/fnbeh.2018.00028)
Supplement: FIGURE S2 — Increased frequency of bite-marks and injuries from fighting in mice in groups containing one or more Cdkn1c-overexpressing mice. There were significantly more signs of severe in-cage fighting (fresh cuts along flanks or in ano-genital region observed on at least one occasion) in cages containing animals over-expressing Cdkn1c and their wild-type cage-mates compared to cages of Cdkn1cBAClacZ animals and their wild-type cage-mates. Within groups of Cdkn1cBACx1 and their wild-type cage-mates signs of severe fighting were not different by genotype. [file Image_2.PDF]

Territorial behaviour and social stability in the mouse require correct expression of imprinted *Cdkn1c*

Gráinne I. McNamara, Rosalind M. John & Anthony R. Isles.

Figure S2

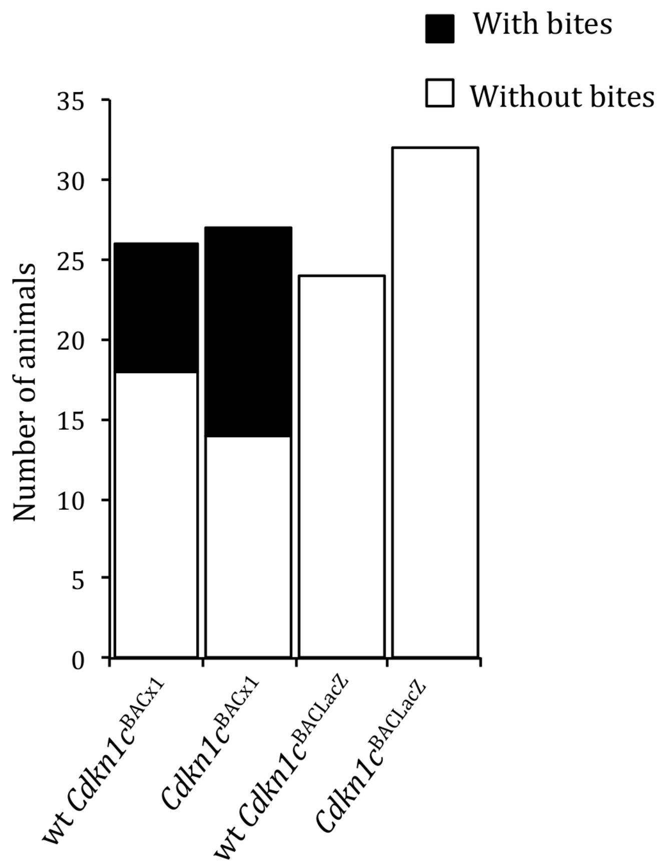

Figure S2 Increased frequency of bite-marks and injuries from fighting in mice in groups containing one or more *Cdkn1c*-overexpressing mice. There were significantly more signs of severe in-cage fighting (fresh cuts along flanks or in ano-genital region observed on at least one occasion) in cages containing animals over-expressing *Cdkn1c* and their wild-type cage-mates compared to cages of *Cdkn1c*^{BAClacZ} animals and their wild-type cage-mates. Within groups of *Cdkn1c*$^{\mathrm{BACx1}}$ and their wild-type cage-mates signs of severe fighting were not different by genotype.
