# Supplementary material for: Territorial Behavior and Social Stability in the Mouse Require Correct Expression of Imprinted Cdkn1c
Source: Front Behav Neurosci. 2018 Feb 26;12:28. doi: 10.3389/fnbeh.2018.00028 (PMC5834910; doi:10.3389/fnbeh.2018.00028)
Supplement: FIGURE S3 — Wild type animals increased scent marking toward Cdkn1cBACx1 but not Cdkn1cBACLacZ animals. Average marking per individual to by a wt cagemate of Cdkn1cBACx1 (green) or a wt cagemate of Cdkn1cBACLacZ (blue) to either a wt or tg cagemate. [file Image_3.PDF]

Territorial behaviour and social stability in the mouse require correct expression of imprinted *Cdkn1c*

Gráinne I. McNamara, Rosalind M. John & Anthony R. Isles.

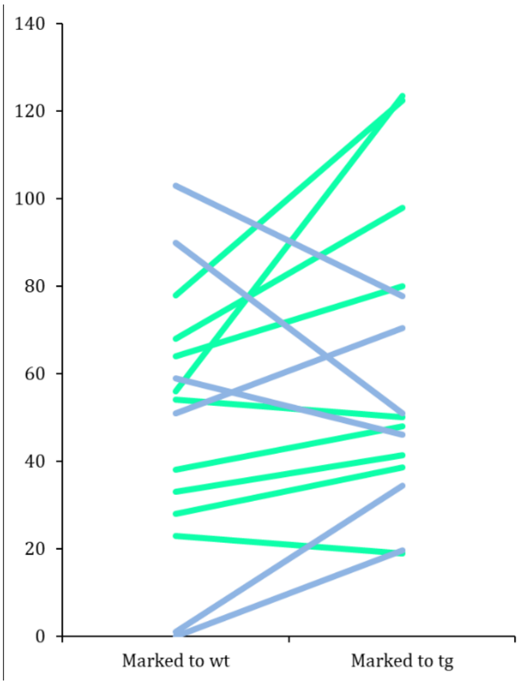

Figure S3. WT animals increased scent marking towards *Cdkn1c*^{BACx1} but not *Cdkn1c*^{BACLacZ} animals.

Average marking per individual to by a wt cagemate of *Cdkn1c*^{BACx1} (green) or a wt cagemate of *Cdkn1c*^{BACLacZ} (blue) to either a wt or tg cagemate.
